# p53, miR-34a and EMP1—Newly Identified Targets of TFF3 Signaling in Y79 Retinoblastoma Cells

**DOI:** 10.3390/ijms20174129

**Published:** 2019-08-24

**Authors:** Maike Busch, Stefan Klein, Jan Große-Kreul, Oliver Scheiner, Klaus Metz, Harald Stephan, Nicole Dünker

**Affiliations:** 1 Institute of Anatomy II, Department of Neuroanatomy, Medical Faculty, University of Duisburg-Essen, 45122 Essen, Germany; 2 Institute of Pathology, Medical Faculty, University of Duisburg-Essen, 45122 Essen, Germany; 3 Division of Haematology and Oncology, Children’s Hospital, University of Duisburg-Essen, 45122 Essen, Germany

**Keywords:** TFF3, EMP1, p53, miR34a, retinoblastoma, chemotherapy, tumor formation, CAM assay

## Abstract

Trefoil factor family peptide 3 (TFF3) is supposed to have tumor suppressive functions in retinoblastoma (RB), but the functional pathway is not completely understood. In the study presented, we investigated the downstream pathway of TFF3 signaling in Y79 RB cells. Results from pG13-luciferase reporter assays and western blot analyses indicate induced p53 activity with an upregulation of miR-34a after TFF3 overexpression. Expression levels of the predicted miR-34a target epithelial membrane protein 1 (EMP1) are reduced after TFF3 overexpression. As revealed by WST-1 assay, BrdU, and DAPI cell counts viability and proliferation of Y79 cells significantly decrease following EMP1 knockdown, while apoptosis levels significantly increase. Opposite effects on Y79 cells’ growth could be shown after EMP1 overexpression. Caspase assays showed that EMP1 induced apoptosis after overexpression is at least partially caspase-3/7 dependent. Colony formation and soft agarose assays, testing for anchorage independent growth, revealed that EMP1 overexpressing Y79 cells have a significantly higher ability to form colonies. In *in ovo* chicken chorioallantoic membrane (CAM) assays inoculated EMP1 overexpressing Y79 cells form significantly larger CAM tumors. Moreover, miR-34a overexpression increases sensitivity of Y79 cells towards RB chemotherapeutics, however, without involvement of EMP1. In summary, the TFF3 signaling pathway in Y79 RB cells involves the activation of p53 with downstream induction of miR-34a and subsequent inhibition of EMP1.

## 1. Introduction

The retinal tumor retinoblastoma (RB) is the most common primary intraocular tumor in childhood with an incidence of 1:16,000 livebirth [1]. Retinoblastoma arises in the developing retina as a part of the central nervous system (CNS) [2]. The molecular mechanisms underlying RB development have been intensively studied during the last decades and it has been postulated to be a multi-step process of progression from normal retinal tissue towards RB cells [3]. Retinal cells homozygous for RB1 loss can form benign tumors called retinomas, but further genomic changes are needed to form retinoblastoma [4]. Beside several potential target genes, MYCN amplifications [5] and dysregulated miRNAs [6] play a role in RB development. Untreated RB will grow and extend beyond the eye and undergo metastatic spread, commonly into regional lymph nodes, bone and CNS [2].

Treatment of RB ranges from enucleation of the affected eye to eye salvage strategies including focal therapy (laser, cryotherapy), brachytherapy and/or chemotherapy. Chemotherapy can be applied systemically, commonly using an intra-arterial [7] and/or intravitreal VEC (vincristine, etoposide, and carboplatin) injection of chemotherapeutics [8]. Chemotherapeutic treatment of RB is, however, limited not only by drug-related side effects, but also by developing drug resistances. Therefore, developing strategies to improve RB therapies for instance by sensitizing RB cells towards chemotherapeutics is a major challenge.

Recent studies by our group suggest that trefoil factor family (TFF) peptides might be potential new candidates for supplementary therapeutic options for retinoblastoma along with common chemotherapy. Three TFF peptides, TFF1, TFF2, and TFF3, have been identified in mammals so far (reviewed in refs. [9,10,11,12,13,14]). They are characterized by a clover leaf—like disulfide structure, the so-called TFF domain. TFFs are normally expressed in the gastrointestinal tract with functions in protection and maintenance of epithelial surfaces [10]. They are, however, also expressed in the central nervous system, in human ocular tissues and in the murine retina (for review see [13]). Previous studies by our group revealed that only TFF3, but not TFF1 is expressed in the healthy human retina [15]. Additionally, TFF peptides are either overexpressed or missing in different human tumor entities and tumor cell lines, e.g., gastric, breast, and ovarian cancer and retinoblastoma cells [13]. Furthermore, *TFF3* expression correlates with the tumor grade in hepatocellular carcinoma [16] and is a marker for poor prognosis in gastric carcinoma [17]. We could demonstrate that the expression of *TFF3* in retinoblastoma cell lines is regulated epigenetically [18] and that forced *TFF3* expression leads to reduced RB tumor growth, viability and tumorigenicity as well as enhanced caspase-dependent apoptosis induction in human RB cell lines [19].

However, the downstream targets of TFF3 signaling during RB development and progression have not been investigated so far. Soutto et al. [20] showed that TFF1 activates p53 in gastric epithelial cell lines. Our group confirmed the activation of p53 in *TFF1* overexpressing RB cell lines [21], but it still remained to be determine whether this signaling cascade also applies for TFF3. MicroRNA 34a (miR-34a) has been found to be a direct transcriptional target of p53 with canonical p53 binding sites in its promotor region [22]. Moreover, in RB cells overexpression of miR-34a leads to enhanced chemosensitivity against the common RB chemotherapeutics vincristine, etoposide and carboplatin [23]. Moreover, the integral membrane glycoprotein epithelial membrane protein 1 (*EMP1*) is a predicted target gene of miR-34a. EMP1 has been shown to be regulated by miR-34a in RB cell lines [24] and is supposed to be a potential biomarker for tumor diagnosis and prognosis of several cancers [25]. Thus, we hypothesized that EMP1 might be a downstream target of TFF3 mediating its anti-proliferative and pro-apoptotic effects in RB cells.

In the study presented, we set out to prove that p53, miR-34a and epithelial membrane protein 1 (*EMP1*) are downstream targets of TFF3 signaling in the retinoblastoma cell line Y79.

## 2. Results

### 2.1. TFF3 Overexpression Activates p53 and miR-34a and Inhibits EMP1 Expression

**Figure 1:**
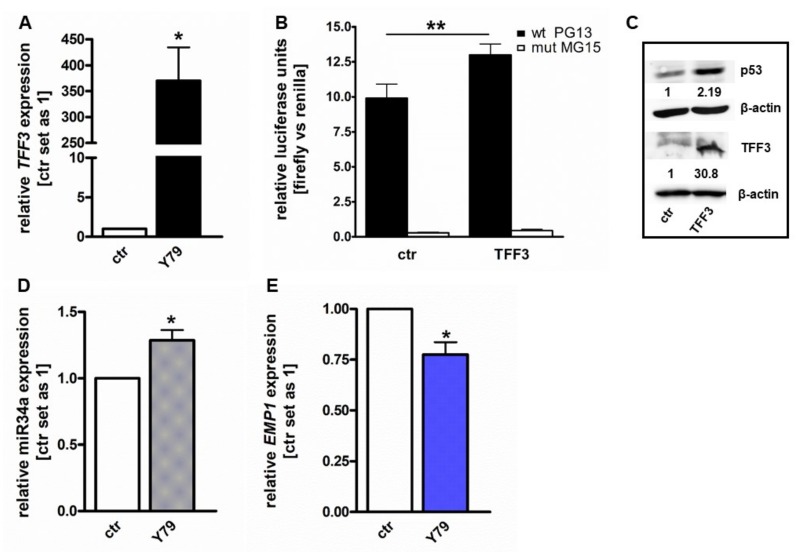
TFF3 overexpression enhances p53 transcriptional activity and protein expression and increases miR-34a expression with downstream reduction of *EMP1* in Y79 RB cells. (**A**) Quantitative Real-time PCR confirmation of *TFF3* lentiviral overexpression (Trefoil factor family peptide 3 (TFF3)) in Y79 cells compared to control cells (ctr). (**B**) Luciferase assays were performed with Y79 cells transiently transfected with *TFF3* or empty vector control (ctr) in addition to wild-type PG13-Luc (wt PG13) or mutant MG15-Luc (mut MG15). Forced TFF3 expression leads to an increased luciferase signal upon p53 promotor activation in Y79 cells. (**C**) Western blot analysis showing increased p53 and TFF3 protein levels after TFF3 overexpression (TFF3). The indicated intensity ratios of p53 and TFF3 protein levels relative to β-actin levels were calculated using ImageJ software. (**D**,**E**) Quantitative real-time PCR analysis of miR-34a and *EMP1* expression levels in Y79 cells compared to control cells after lentiviral TFF3 overexpression (ctr). Values are means of at least 3 independent experiments ± SEM. * *p*-value < 0.05, ** *p*-value < 0.01; statistical differences compared to the control group calculated by Student’s *t*-test or one-way ANOVA and Newman-Keuls post test.

We performed a lentiviral overexpression of TFF3 in Y79 RB cells (Figure 1A) and analyzed different potential target genes involved in TFF3 signaling to investigate the underlying mechanisms of TFF3’s tumor-suppressive functions. We first analyzed the transcriptional activation of p53 by *luciferase* reporter assay. For this purpose, Y79 cells were transiently transfected with pG13-*Luc* (with p53 binding site) and the non-responsive reporter pG15-*Luc* (with a mutant p53 binding site) along with TFF3 or an empty vector control. Compared to control cells the relative *luciferase*-activity of the p53 responsive reporter was significantly increased in TFF3 overexpressing cells (Figure 1B). western blot analysis revealed a 2-fold increase in p53 expression in TFF3 overexpressing Y79 cells (Figure 1C).

Next, we analyzed the expression of miR-34a after TFF3 overexpression. Quantitative Real-time PCR analysis revealed significantly increased levels of miR-34a expression after *TFF3* overexpression in Y79 cells (Figure 1D). Moreover, Real-time PCR analyses of Y79 RB cells revealed significantly reduced *EMP1* expression levels following TFF3 overexpression in comparison to control cells (Figure 1E). All RB cell lines except for Rbl13 and all primary patients’ tumor samples analyzed exhibit higher endogenous miR34a expression levels and lower EMP1 expression levels compared to a healthy human retina pool (Figure S1).

### 2.2. EMP1 Knockdown Inhibits Growth and Induces Apoptosis in Y79 Cells

**Figure 2:**
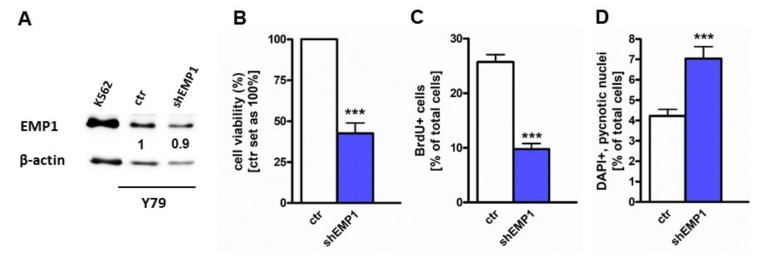
Epithelial membrane protein 1 (EMP1) knockdown leads to reduced cell viability and proliferation and induces apoptosis in Y79 RB cells. (**A**) Western blot data confirmed decreased EMP1 protein levels after EMP1 knockdown (shEMP1) in Y79 cells. The CML cell line K562 served as an EMP1 positive control, ß-actin as a loading control. (**B**,**C**) Stably EMP1 knockdown Y79 RB cells (shEMP1) showed significantly decreased viability and proliferation levels compared to control cells (ctr) as revealed by (**B**) WST-1 assays and (**C**) BrdU stains. (**D**) EMP1 knockdown Y79 cells (shEMP1) displayed a significantly increased apoptosis rate compared to control cells (ctr) as revealed by DAPI stains. Values are means of 3 independent experiments ± SEM. *** *p*-value < 0.001 statistical differences compared to the control group calculated by Student’s *t*-test.

A previous study by our group demonstrated that TFF3 overexpression reduces viability and proliferation and enhances apoptosis in human RB cell lines [19]. Here we demonstrate that EMP1 levels are downregulated after TFF3 overexpression (Figure 1E). Hypothesizing that EMP1 triggers the effects seen after TFF3 overexpression, we knocked EMP1 down in order to prove that reduced EMP1 levels provoke the same effects as TFF3 overexpression. EMP1 knockdown was confirmed by Real-time PCR (Supplementary Figure S2A) and western blot analysis (Figure 2A). Y79 cells with reduced EMP1 expression levels exhibited significantly lower cell viability (Figure 2B) and displayed significantly decreased proliferation levels as revealed by BrdU cell counts (Figure 2C). Moreover, after EMP1 knockdown a significant increase in apoptosis levels was detectable (Figure 2D).

### 2.3. EMP1 Overexpression Induces Growth and Inhibits Apoptosis in Y79 Cells

As EMP1 knockdown results in a strong anti-proliferative effect, hampering any further detailed in vitro and subsequent in vivo analyses, we analyzed the effects of EMP1 overexpression in Y79 cells, expecting to reveal the opposite effects.

**Figure 3:**
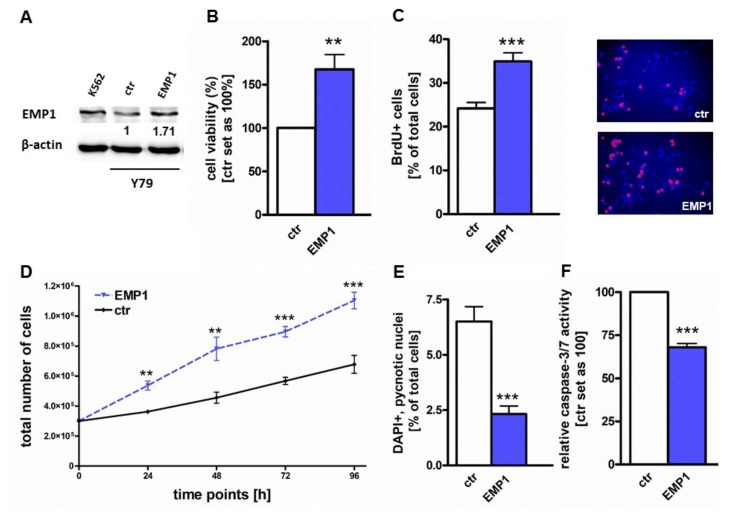
EMP1 overexpression leads to increased cell viability and proliferation and induces caspase-3/7 dependent apoptosis in Y79 RB cells. (**A**) Western blot data confirmed increased EMP1 protein levels after EMP1 overexpression (EMP1) in Y79 cells. The CML cell line K562 served as an EMP1 positive control, ß-actin as a loading control. (**B**,**C**) Stably EMP1 overexpressing Y79 RB cells (EMP1) showed significantly increased viability and proliferation levels compared to control cells (ctr) as revealed by (**B**) WST-1 assays and (**C**) BrdU stains. red: BrdU-labeled cells; blue: DAPI counterstaining (**D**) Growth curve analysis of EMP1 overexpressing Y79 RB cells showed a significant increase in cell growth rates. (**E**) EMP1 overexpressing Y79 cells (EMP1) displayed a significantly reduced apoptosis rate compared to control cells (ctr) as revealed by DAPI stains. (**F**) Caspase-3/7 activity was significantly reduced after EMP1 overexpression in Y79 RB cells (EMP1) compared to control cells (ctr). Values are means of at least 3 independent experiments ± SEM. ** *p*-value < 0.01; *** *p*-value < 0.001 statistical differences compared to the control group calculated by Student’s *t*-test.

EMP1 overexpression was confirmed by Real-time PCR (Supplementary Figure S2B) and western blot analysis (Figure 3A). EMP1 overexpressing Y79 cells exhibited a significantly higher cell viability (Figure 3B) and displayed significantly increased growth as revealed by BrdU cell counts (Figure 3C) as well as growth curve analyses (Figure 3D). Moreover, after EMP1 overexpression a significant decrease in apoptosis levels was detectable (Figure 3E), which turned out to be caspase3/7-dependent (Figure 3E).

### 2.4. EMP1 Overexpression Increases Tumorigenicity of Y79 Cells In Vitro and In Vivo

**Figure 4:**
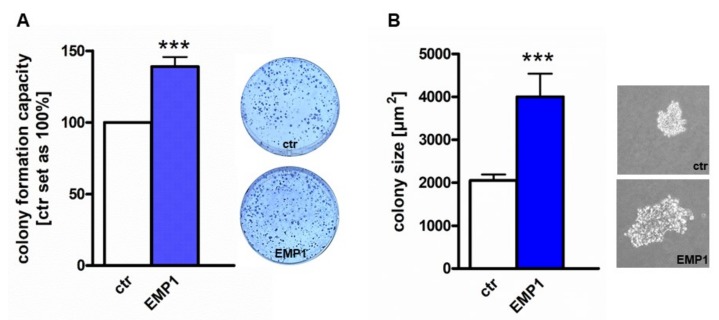
Effect of stable EMP1 overexpression on RB cell colony formation and anchorage independent growth. (**A**) Quantification of colony formation assays (CFA) showing a significant higher capacity of EMP1 overexpressing Y79 RB cells to form colonies (EMP1) compared to control cells (ctr). (**B**) Quantification of anchorage independent growth capacity of EMP1 overexpressing Y79 RB cells (EMP1) compared to control cells (ctr) as revealed by soft agarose assay. All photographs are taken 3 weeks after seeding EMP1 overexpressing and control Y79 RB cells. Values are means of at least 3 independent experiments ± SEM. *** *p*-value < 0.001; statistical differences compared to the control group calculated by Student’s *t*-test.

Colony formation and soft agarose assays indicated a significant higher ability of EMP1 overexpressing Y79 cells to form colonies (Figure 4A) and to grow anchorage independently (Figure 4B).

**Figure 5:**
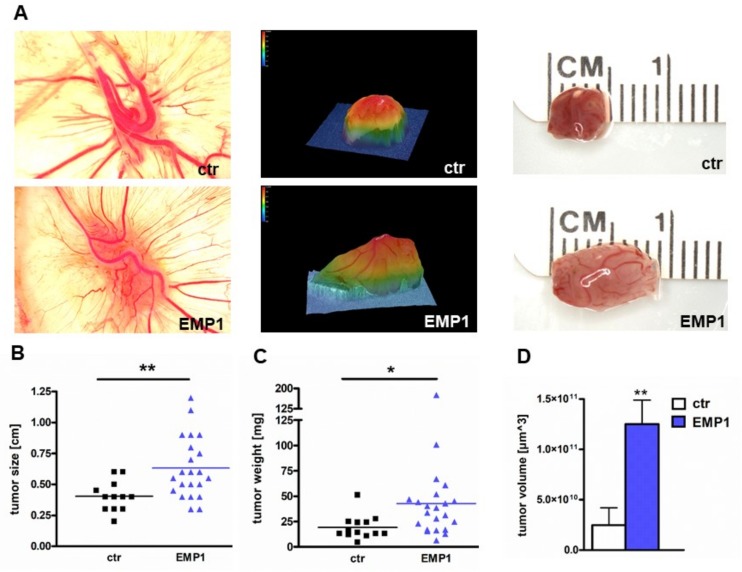
Stable, lentiviral EMP1 overexpression increases tumor formation capacity of Y79 RB cells. (**A**) Photographs of CAM tumors in situ (left column), 3D tumor volume (middle column) and ruler measurements (in cm) of excised tumors (right column) revealing that tumors forming in the upper CAM after grafting EMP1 overexpressing Y79 RB cells were significantly larger compared to those developing from control cells (ctr). (**B**,**C**) Quantification of CAM assays by (**B**) tumor size, (**C**) tumor weight and (**D**) tumor volume. Values are means from at least 3 independent experiments (except for tumor volume which was measured exemplarily in one experimental setting) ± SEM. * *p*-value < 0.05, ** *p*-value < 0.01 statistical differences compared to the control group calculated by Student’s *t*-test.

To investigate whether EMP1 influences RB cells’ tumor growth, we used the chicken chorioallantoic membrane (CAM) as an in vivo model. Y79 cells stably overexpressing EMP1 and control cells were inoculated onto the CAM of 10-day old chicken embryos. Photo-documentation of the tumors developing from Y79 cells inoculated onto the CAM (Figure 5A) and quantification of tumor size, weight and volume revealed that EMP1 overexpressing RB cells develop significantly larger tumors (Figure 5B) than control cells, exhibiting higher weights (Figure 5C) and volumes (Figure 5D). There are no significant changes in the number of developing tumors between EMP1 overexpressing cells and control cells (data not shown).

### 2.5. MiR-34a and EMP1 Overexpression Enhances Chemosensitivity of Y79 RB Cells

As a previous study revealed that overexpression of miR-34a in RB cells leads to enhanced chemosensitivity against chemotherapeutics [23] and EMP1 has been shown to be regulated by miR-34a in RB cell lines [24], we analyzed the effect of miR-34a and EMP1 overexpression on Y79 cells’ sensitivity towards the common RB chemotherapeutics etoposide, vincristine and cisplatin.

Compared to untreated controls, apoptosis levels of untreated miR34a overexpressing Y79 cells significantly increased after miR-34a overexpression. Additional treatment with low doses (0.01 µM) of the chemotherapeutics vincristine, etoposide, and cisplatin significantly augmented this effect (Figure 5A) indicating that miR34a overexpression increases Y79 cells’ chemosensitivity towards VEC chemotherapeutics. Cell viability assays confirmed this notion as not only the viability of miR34 overexpressing Y79 cells significantly decreased, but subsequent application of VEC chemotherapeutics yet resulted in an additional decrease of viability levels except for vincristine treatment (data not shown).

**Figure 6:**
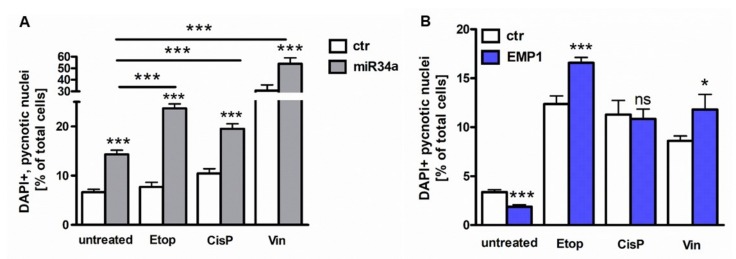
MiR-34a and EMP1 overexpression leads to enhanced chemosensitivity. (**A**) Overexpression of miR-34a in Y79 RB cells leads to significantly increased apoptosis levels compared to control cells (ctr). Additional treatment with etoposide (Etop), cisplatin (CisP), or vincristine (Vin) significantly elevates the apoptosis levels compared to untreated miR-34a overexpressing Y79 RB cells. (**B**) Overexpression of EMP1 in Y79 RB cells leads to significantly decreased apoptosis levels compared to control cells (ctr). Additional treatment with etoposide (Etop) and vincristine (Vin) leads to higher apoptosis levels compared to untreated EMP1 overexpressing Y79 RB cells. Additional treatment with cisplatin (CisP) did not change the apoptosis level compared to untreated EMP1 expressing Y79 RB cells. Values are means of at least 3 independent experiments ± SEM. * *p*-value < 0.05, *** *p*-value < 0.001; statistical differences compared to the control group calculated by one-way ANOVA and Newman-Keuls post test.

In order to test if miR-34a induced chemosensitivity is mediated via downstream regulation of EMP1, we analyzed the effect of EMP1 overexpression on Y79 cells’ resistance towards commonly used RB chemotherapeutics. As shown before (Figure 3E), apoptosis levels of untreated Y79 cells significantly decreased after EMP1 overexpression (Figure 6B). In EMP1 overexpressing cells, however, additional treatment with high doses of VEC chemotherapeutics resulted in significantly enhanced apoptosis levels compared to respective control cells, except for cisplatin treatment (Figure 6B), indicating that contrary to our expectations, EMP1 overexpression does not increase the resistance of Y79 cells towards cytostatic drugs, but likewise increases chemosensitivity of RB cells.

## 3. Discussion

The role of TFFs in cancer progression of different tumor entities has been largely described in the literature [26,27]. Especially the function of TFF3 is controversially discussed as it is considered as a potential oncogenic factor or a tumor suppressor gene depending on the tissue investigated [26,28]. Ectopic TFF3 expression in cancer tissues like gastric, pancreatic, hepatocellular, colon, and breast cancer is linked to enhanced cell proliferation and survival as well as increased invasion and migration [13,29]. By contrast, in most thyroid tumors and in retinoblastoma TFF3 expression is decreased [19,30]. We demonstrated previously that this reduction in *TFF3* expression in RB cell lines is regulated epigenetically [18]. In addition, we could recently show that *TFF3* overexpression in four different RB cell lines has anti-proliferative and pro-apoptotic effects in vitro and leads to reduced tumor growth in vivo, supporting the notion of *TFF3* as a tumor suppressor in retinoblastoma [19]. In the present study, we investigated the mechanisms underlying *TFF3* mediated effects exemplarily in the RB cell line Y79. In accordance with findings of Soutto et al., who showed a TFF1 mediated p53 activation in gastric cancer cells [20], our group recently demonstrated that in RB cell lines *TFF1* overexpression is likewise related to p53 activation [21]. We therefore hypothesized that p53 activation in RB cells might also be induced by *TFF3* overexpression and the study presented proved this hypothesis right. We could show increased p53 protein levels and a significant activation of p53 in *TFF3* overexpressing Y79 RB cells. Which binding factors are influenced by TFF3 resulting in p53 induction, needs to be revealed in future studies.

Next, we analyzed the expression levels of the known p53 microRNA target miR-34a [31], which was concordantly upregulated after *TFF3* overexpression. Ectopic re-expression of miR-34a was already correlated to apoptosis and growth inhibition in multiple myeloma cells [32] and Dalgard et al. showed that miR-34a functions as a tumor suppressor in RB cells, highlighting it as a potential therapeutic target [24]. In line with these findings treatment with miR-34a mimics in lung cancers has been evaluated as a replacement therapy as miR-34a reduces lung tumor growth [22]. These effects are at least partially mediated by the direct post-transcriptional repression of postulated miR-34a target genes. Among other oncogenic substrates of miR-34a *EMP1* is one of the predicted targets, regulated by miR-34a overexpression in RB cells lines [24]. The integral membrane glycoprotein EMP1 is commonly expressed in epithelial tissues of the gastrointestinal tract, skin, lungs, and brain [33,34]. Alterations of *EMP1* expression have been linked to a variety of human cancers like gliomas, gastric cancer as well as in oral, laryngeal, scirrhous gastric, and esophageal cancers [35,36,37,38]. However, the function of EMP1 is controversially discussed in the literature. In most cancers, tumor suppressive functions for EMP1 have been described like inhibition of cell growth and metastasis by induction of apoptosis and prevention of angiogenesis [39]. In these tumor entities loss of EMP1 expression correlates with poor prognosis. By contrast, EMP1 silencing in T-ALL cell lines leads to increased apoptosis levels, reduced cell survival, and sensitized the cells towards prednisolone treatment [40]. In these cancers high endogenous EMP1 expression correlates with poor patients’ outcome. In line with these findings, silencing EMP1 in chondrocytes inhibits cell proliferation [41].

In the present study, we detected a significant reduction in EMP1 expression levels after *TFF3* overexpression in Y79 cells, accompanied by *miR-34a* upregulation. Thus, we set out to verify if EMP1 knockdown will lead to the same functional effects as TFF3 overexpression. We could show that knocking down EMP1 in Y79 cells indeed mimics TFF3 overexpression effects leading to reduced cell viability and proliferation and increased apoptosis levels. In order to show the tumor inductive potential of EMP1 in RB cells, we additionally overexpressed EMP1 in Y79 cells expecting to see the opposite effects following *TFF3* overexpression, namely higher cell viability and proliferation levels as well as decreased cell death levels. Indeed, EMP1 overexpression in Y79 cells resulted in an induction of cell viability, concomitantly increased proliferation rates and reduced caspase3/7 dependent apoptosis levels. Additionally, colony formation capacity and anchorage independent growth were increased after EMP1 overexpression compared to control cells. The in vitro experiments were confirmed in vivo as EMP1 overexpressing Y79 cells displayed a higher tumorigenicity, resulting in significantly larger CAM tumors.

In line with our results, Johnson et al. showed a correlation between *TFF1* expression and *EMP1* regulation. Gene expression analyses of *Tff1-/-* mice revealed that EMP1 is significantly upregulated when *Tff1* expression is lacking [34]. By contrast, our present study revealed that compared to healthy human retina tissue the endogenous EMP1 expression seems to be reduced in different RB cell lines and primary RB tumors lacking TFF3 expression (Figure S1A). This might be an effect of the relatively high endogenous miR-34a expression levels in RB cells (Figure S1B). MiR-34a is currently considered to be a promising therapeutic agent, which increases chemosensitivity and decreases aggressiveness of cancer cells and as a potential prognostic biomarker for treatment strategies. Several publications indicate a role for miR-34a in chemotherapy response in a number of tumor models including NSCLC, HCC, breast cancer, bladder cancer, colorectal cancer, prostate cancer, and head and neck squamous cell carcinoma (for review see: [31]). A higher miR-34a expression increases the sensitivity to cisplatin for lung and bladder cancer cells [42,43] as well as for RB cells to treatment with vincristine, etoposide, or carboplatin [23]. In the study presented, we confirmed an increase in chemosensitivity to the RB VEC chemotherapeutics vincristine, etoposide and cisplatin in miR-34a overexpressing Y79 cells. We also addressed the question if the chemosensitizing effects are exclusively induced by miR-34a or if the effects could also be mediated by downstream regulation of EMP1. In this respect, we overexpressed EMP1 in Y79 cells and additionally treated them with VEC chemotherapeutics in order to prove if a higher EMP1 expression leads to chemoresistance. However, in comparison to control cells no augmented chemoresistance was detectable after combined EMP1 overexpression and VEC treatment, but, contrarily, an increase in apoptosis levels was observed after treatment with etoposide and vincristine following EMP1 overexpression. The enhanced sensitivity towards cytostatic drugs observed in EMP1 overexpressing Y79 cells is most likely mediated by the pro-proliferative effects of EMP1 overexpression itself, as rapidly dividing cells are potentially more susceptible to the topoisomerase II inhibitor etoposide and vincristine-mediated tubulin binding. Thus, in RB cells the chemosensitizing effects of miR-34a are not mediated by regulation of EMP1.

In summary, we could demonstrate that the overexpression of *TFF3* in Y79 RB cells leads to an activation of p53 and an upregulation of *miR-34a,* a direct target of p53. Moreover, the expression of the predicted miR34a target EMP1 was reduced after TFF3 overexpression. Taken together, our data strengthen the tumor suppressive function of *TFF3* in retinoblastoma cells and emphasize TFF3’s downstream signaling components miR-34a and EMP1 as potential targets for adjuvant RB therapy and drug development strategies.

## 4. Material and Methods

### 4.1. Human Retina and Retinoblastoma Samples

Post mortem human retina samples from cornea donors and retinoblastoma samples were used for comparative expression studies. The Ethics Committee of the Medical Faculty of the University of Duisburg-Essen approved the use of human retina (approval # 06-3021, 22 March 2006) and retinoblastoma samples (approval # 14-5836-BO, 07 May 2014) for research conducted in the course of the study presented and written informed consent has been obtained from patients’ relatives or parents.

### 4.2. Cell Culture

The human retinoblastoma cell lines Y79 [44,45], originally purchased from the Leibniz Institute DSMZ (German Collection of Microorganisms and Cell Cultures) were kindly provided by Dr. H. Stephan. All RB cell lines used in this study were cultivated as described previously [45].

Human embryonic kidney cells (HEK293-T) were grown as adherent cell cultures in DMEM with 10% FBS, 4 mM L-glutamine, 100 U penicillin/mL and 100 µg streptomycin/mL at 37 °C, 5% CO_2_, and 95% humidity.

### 4.3. Treatment with Chemotherapeutics

To investigate chemosensitivity, miR34a or EMP1 overexpressing Y79 cells and control cells transfected with an empty vector control were treated individually with three different chemotherapeutics (vincristine, etoposide, and cisplatin) and compared to untreated cells. Twenty-four hours before the transient transfection with miR-34a, 2 × 10^5^ Y79 cells/mL were seeded in growth medium without antibiotics. Twenty-four hours after transient transfection, Y79 cells were treated with low effective doses (0.01 µM) of vincristine, etoposide or cisplatin for 72 h, identified by previous concentration analyses. Y79 cells stably transduced with EMP1 lentiviral particles were treated with highly effective doses of 2.5 nM vincristine, 1 µM etoposide or cisplatin for 72 h as previously described by Liu et al. [23]. After 72 h of treatment with chemotherapeutics cell viability was analyzed by WST-1 assay and apoptosis via DAPI cell counts.

### 4.4. Plasmids and Lentiviral Expression Vectors

To generate a lentiviral *EMP1* expression vector (pLenti CMV_*EMP1*), the human *EMP1* cDNA sequence was amplified from the RC208410 vector (Origene) using the forward primer 5′-CGAATTCTATGTTGGTATTGCTGGCTGG-3′ and the reverse primer 5′-CGCTCGAGTTATTTCTTTCTCAGGACCAG-3′ containing *Eco*RI and *Xho*I restriction sites (underlined). The PCR product was cloned into the pCRII-TOPO vector (Invitrogen), excised by digestion with *EcoRI* and *Xho*I and ligated into the pENTR4 vector, digested with the same restriction enzymes. *EMP1* was finally cloned into the pLenti CMV Puro Dest vector (Addgene) by the use of a Gateway LR Clonase II Enzyme Mix (Invitrogen), following the manufacturer’s protocol. The “Mission shRNA Plasmid DNA” with a pLKO.2-puro backbone used in the EMP1 knockdown experiments was purchased from Sigma-Aldrich (St. Louis, MI, USA), (shEMP1 *clone*TRCN0000117946). The non-mammalian shRNA control pPRIME-CMV-Neo-FF3 (p234) containing a targeting hairpin sequence against *firefly-luciferase* was used in all transduction experiments [46].

To generate a miR34a expression vector (pSG5-miR34a), the human miR34a sequence was amplified from cDNA of HEK293T cells by RT-PCR using the forward primer 5′-CGGAATTCGCATCCTTTCTTTCCTCCCC-3′ and the reverse primer 5′-CGGGATCCGGGCATCTCTCGCTTCATCT-3′ containing *EcoRI* and *BamHI* restriction sites (underlined). The PCR product was cloned into the pCRII-TOPO vector (Invitrogen, Karlsruhe, Germany), excised by digestion with EcoRI and BamHI and ligated into the pSG5 vector digested with the same restriction enzymes. All vector constructs were verified by sequencing.

### 4.5. TFF3 and EMP1 Overexpression and EMP1 Knockdown in Y79 Retinoblastoma Cells

For transient *TFF3* overexpression, Y79 RB cells were transfected as previously described by our group [19].

For virus production, HEK293T cells were transfected as described previously [45] with each of the following plasmid DNAs: packaging vector pczVSV-G [47], pCD NL-BH [47], and either pLenti CMV_TFF3, pLenti CMV_EMP1, shEMP1 *clone*TRCN0000117946 or pPRIME-CMV-Neo-FF3 (p234).

Experimental conditions for the lentiviral transductions were the same as described previously [19]. In addition to *TFF3*-coding lentiviral particles *EMP1*-coding particles were used.

### 4.6. RNA Extraction and Quantitative Real-Time PCR

RNA isolations from RB cells were performed using the miRNeasy Kit (Qiagen, Hilden, Germany) and the miRNeasy FFPE Kit (Qiagen), respectively.

For quantitative Real-time PCR analyses cDNA was synthesized with the QuantiTect Reverse Transcription Kit (Qiagen) following the manufacturer’s protocol and the following human Taqman Gene Expression Assays (Applied Biosystems, Dreieich, Germany) were used: *EMP1* (Hs0060855_m1) and *18S* (Hs99999901_s1). The latter was used as an endogenous control. In RT-qPCR reactions, conducted in duplicates, 20 µL of a TaqMan Universal PCR Master Mix (Applied Biosystems) was used and the samples were run in the following program: 50 °C for 2min, 95 °C for 10 min, 95 °C for 15 s for 40 cycles, 60 °C for 60 s.

After cDNA synthesis, we used the miScript PCR Starter Kit (# 2181193; Qiagen) for miRNA RT-qPCR analyses, following the instructions of the manufacturer. For the quantification of mature miRNAs, a designated miScript HiSpec buffer was used together with specific primers for miR-34a (5′-TGGCAGTGTCTTAGGTGGTTGT-3′) and 5.8S RNA (5′-CTACGCCTGTCT GAGCGTCGCTT-3′) as endogenous control. The reactions were performed in duplicates using a 7300 Real-Time PCR System (Applied Biosystems).

### 4.7. Western Blotting

After washing in PBS, cells were lysed for 60 min at 4 °C in RIPA buffer plus additives (see: [15]) and cleared by a 10 min centrifugation step at 14,000 rpm and 4 °C. Equal amounts of protein extracts were separated on a 12% SDS-PAGE and transferred onto nitrocellulose membranes. Primary antibodies used (incubated overnight at 4 °C): EMP1 (1:1000): ab230445, Abcam; p53 (1:400): sc-71817, Santa Cruz; TFF3 (1:500): TA307376, Origene; ß-actin (1:1000): #4967, Cell Signalling. Secondary antibodies used: HRP-conjugated goat-anti-rabbit or rabbit-anti-mouse antibody (1:10,000): P0448 and P0260; DAKO. Signals were developed by the Western Bright Chemiluminescence Reagent (*Advansta*).

### 4.8. Cell Viability

To determine cell viability, 3 × 10^5^ cells in 500 µL medium were seeded in a 24-well plate. After 72 h of incubation, 100 µL cell suspensions were seeded in two triplicates into a 96 well plate. After adding 10 µL of a WST (water soluble tetrazolium) solution to each well, we incubated the cells at 37 °C for a designated period. The formazan product of viable cells was quantified in a microplate reader at an absorbance of 450 nm.

### 4.9. Growth Kinetic

For growth kinetics analyses in a in 24-well plate format, we seeded 3 × 10^5^ cells in 500 µL supplemented DMEM in triplicates and determined the number of vital cells by manual cells counts every 24 h (5 time points: 0 h, 24 h, 48 h, 72 h, and 96 h) after trypan blue exclusion.

### 4.10. Cell Proliferation and Apoptosis Detection

To determine cell proliferation, 4 h prior to PFA fixation 5 µM BrdU (5-Bromo-2′-deoxyuridine; BrdU; Sigma) were added to the cells. The BrdU signal was revealed by a rat anti-BrdU primary antibody (1:1000; ab6326; Abcam, Cambridge, UK) and visualized by an Alexa Flour 594-labelled goat anti-rat secondary antibody (1:1000; Molecular Probes, Eugene, OR, USA). Changes in cell death levels were determined by manual counts of pycnotic nuclei after 4′, 6-Diamidino-2-phenylindole (DAPI; Sigma) stains.

For each experiment six coverslips were stained and the percentages of proliferating or apoptotic cells were calculated as described previously by our group [45,48].

### 4.11. Caspase-Glo 3/7 Assay

To analyze caspase 3 and 7 cleavage activity after stable EMP1 transduction of Y79 cells a caspase-Glo 3/7 assay (Promega, Madison, WI USA) was used and luminescence was measured. Therefore, 9 × 10^5^ EMP1 overexpressing and control cells were seeded in 300 µL growth medium (24 well plate) and incubated overnight. After 24 h 80 µL of each cell suspension were mixed with 80 µL of caspase-3/7 reagent and seeded in a white 96 well plate for 2 h. Following the manufacturer’s instructions luminescence was measured after 2 h of incubation (Orion II microplate luminometer). The reactions were performed twice in triplicates.

### 4.12. Colony Formation and Soft Agarose Assay

For colony formation assays (CFA) 4000 cells were seeded in 2 mL of DMEM with supplements on poly-D-lysine coated 6 well plates. After three weeks of incubation cells were fixed and stained with a 2.5% crystal violet/10% formalin solution for 30 min at RT. After three washes with water and air drying the colonies were photographed for colony density measurements with ImageJ software. Soft agarose assays were performed as described in detail previously [46]. Colony formation efficiency (%) per visual field was determined by counting colonies and single cells (in triplicates) in five visual fields (10×) per cell line.

### 4.13. CAM Assay

In order to test for changes in tumor formation and migration capacity, *EMP1* overexpressing Y79 cells and control cells were grafted on the chorioallantoic membrane (CAM) mainly following Zijlstra and Palmers protocols [49,50]. Twenty eggs were grafted in at least three independent experiments. Seven days after grafting (E10-17) tumors which formed from the grafted cells were excised, measured and photographed as described previously [19].

### 4.14. Luciferase Assay

TFF3 dependent p53 activity was measured with the p53 responsive reporter PG13-*Luc* (with p53 binding site) and the non-responsive reporter MG15-*Luc* (with a mutant p53 binding site; Addgene [51]) after co-transfection of pCS2+_UBQ_TFF3 or an empty control vector (pCS2+_UBQ). As controls, cells were transfected with a promotor-less pGL3-basic and a pGL3-control *luciferase* vector containing an SV40 promotor and enhancer. All cells were additionally co-transfected with pGL4.75 (*Renilla-Luc*) for normalization. Cells were lysed after 48 h in 1× passive-lysis buffer (Promega) and the *luciferase*-activity was measured with the “Dual Luciferase reporter assay system” (E1910; Promega and Glomax 20/20 luminometer) as described by the manufacturer. The relative p53 *luciferase*-activity was determined as the quotient of *firefly-luciferase* and *Renilla-luciferase*-activity. Analyses were performed in triplicates.

### 4.15. Statistical Analysis

All assays were performed in triplicates. Statistical analyses were performed using GraphPad Prism 6. Data represent means ± SEM of three independent experiments from independent RB cell cultures. Results were analyzed by a Student’s *t*-test or one-way ANOVA and Newman-Keuls post tests and considered significantly different if *p*-value < 0.05 m (*), *p*-value < 0.01 (**) or *p*-value < 0.001 (***). Statistics on the growth curves was performed using a free web interface http://bioinf.wehi.edu.au/software/compareCurves/, which uses the “compare growth curves” function from a statistical modeling package called statmod, available from the “R Project for Statistical Computing”: http://www.r-project.org, previously described elsewhere [52].

## Supplementary Materials

Supplementary materials can be found at https://www.mdpi.com/1422-0067/20/17/4129/s1.
