# Supplementary material for: p53, miR-34a and EMP1—Newly Identified Targets of TFF3 Signaling in Y79 Retinoblastoma Cells
Source: Int J Mol Sci. 2019 Aug 24;20(17):4129. doi: 10.3390/ijms20174129 (PMC6747266; doi:10.3390/ijms20174129)
Supplement: Supplementary file 1 [file ijms-20-04129-s001.pdf]

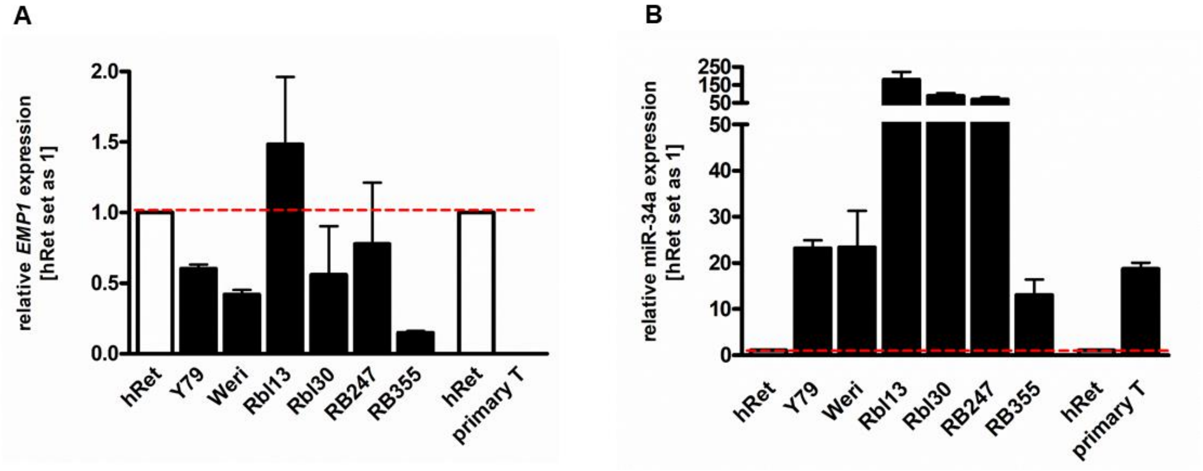

Figure S1. Endogenous *EMP1* and miR-34a expression levels in different RB cell lines and primary tumor samples. (A+B) Quantitative Real-time analysis of *EMP1* and miR-34a expression levels of six different RB cell lines (Y79, Weri, Rbl13, Rbl30, RB247 and RB355) and patients` RB tumors (primary T) compared to a healthy human retina pool (hRet).

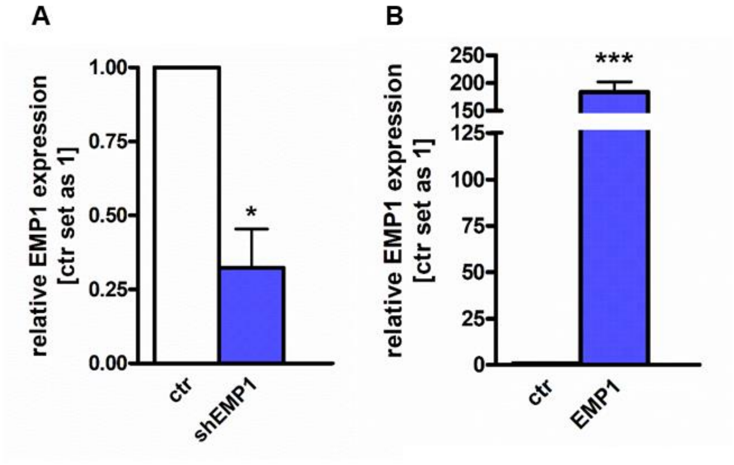

Figure S2. Relative expression level after lentiviral EMP1 knockdown (A) and EMP1 overexpression (B) in Y79 RB cells compared to control cells. Values are means from at least 3 independent experiments ± SEM. ***P-value < 0.001 statistical differences compared to the control group calculated by Student`s *t*-test.
